# Investigation of renal tubular function with newly diagnosed type 1 diabetes mellitus during diabetic ketoacidosis

**DOI:** 10.1186/s13098-024-01506-6

**Published:** 2024-11-20

**Authors:** Naonori Kumagai, Hiroki Takao, Yuta Sudo, Masatoshi Yoshikane, Tomomi Kondoh, Yuji Matsumoto, Haruo Mizuno, Michiaki Abe, Yohei Ikezumi

**Affiliations:** 1 Department of Pediatrics, Fujita Health University School of Medicine, Toyoake, Japan; 2 Department of Education and Support for Regional Medicine, Tohoku University Hospital, Sendai, Japan

**Keywords:** Type 1 diabetes mellitus, Diabetic ketoacidosis, Proximal renal tubular dysfunction, Distal renal tubular acidosis, Fanconi syndrome

## Abstract

**Background:**

Proximal renal tubular dysfunction occurs during diabetic ketoacidosis (DKA) in type 1 diabetes. However, only a few studies have reported on the multiple proximal renal tubular functions simultaneously. Moreover, to the best of our knowledge, distal renal tubular function has not yet been investigated.

**Methods:**

Patients with newly diagnosed type 1 diabetes mellitus were classified into those with DKA and those without DKA, and their proximal and distal renal tubular functions were investigated. The diagnostic criteria for DKA were blood glucose > 200 mg/dL, blood pH < 7.3 or HCO_3_^–^ < 15 mEq/L, and urine ketone body positivity.

**Results:**

Six patients with DKA and five patients without DKA were included. In patients with DKA, urinary β2-microglobulin levels were significantly higher, while blood pH, HCO_3_^–^, and tubular reabsorption of phosphorus were significantly lower than in those without DKA. There were no significant differences in blood glucose, HbA1c, serum phosphorus, urinary N-acetyl-beta-glucosaminidase, and urinary amino acid excretion between patients with and without DKA. Elevated NH_3_ levels and impaired urinary acidification were not observed in patients with and without DKA.

**Conclusions:**

In patients with newly diagnosed type 1 diabetes mellitus complicated with DKA, multiple proximal renal tubular dysfunctions occur simultaneously, suggesting transient Fanconi syndrome. Distal renal tubular acidosis was unlikely. The diagnostic criteria for DKA are appropriate also in the view of proximal renal tubular dysfunction and are considered suggestive of pathophysiological factors that may cause proximal renal tubular dysfunction.

## Introduction

Type 1 diabetes mellitus, which results from damage to pancreatic β cells and absolute insulin deficiency, is the most common cause of diabetes mellitus in children [1]. Children with newly diagnosed type 1 diabetes mellitus often present with diabetic ketoacidosis (DKA) [2]. DKA is a frequent and serious complication of diabetes mellitus characterized by hyperglycemia, ketone body accumulation, and metabolic acidosis, leading to acid–base imbalance and electrolyte abnormalities [3]. Metabolic acidosis, which is an acid–base balance abnormality, consists of both anion gap (AG) metabolic acidosis due to ketone body accumulation and normal AG metabolic acidosis due to HCO_3_^–^ loss [4, 5]. Hyponatremia and hypophosphatemia are commonly observed electrolyte abnormalities.

Elevated urinary β2-microglobulin (β2-MG) levels [6], elevated urinary N-acetyl-beta-glucosaminidase (NAG) levels [6], and increased urinary amino acid excretion have been reported in patients with DKA or poorly controlled type 1 diabetes mellitus [7, 8]. Moreover, it has also been reported that impaired phosphorus reabsorption is a complication of long-term morbidity [6]. It has been reported that at the onset of childhood type 1 diabetes mellitus, some kind of proximal renal tubular damage is observed in all patients with DKA, and even in the patients without DKA, some kind of proximal tubular damage was commonly observed [9]. These findings suggest that proximal renal tubular dysfunction occurs during DKA and is involved in acid–base imbalance and electrolyte abnormalities. However, to the best of our knowledge, only a few studies have reported on the multiple proximal renal tubular functions simultaneously. Moreover, as far as we can tell, distal renal tubular function has not yet been investigated.

In this study, we classified patients with newly diagnosed type 1 diabetes mellitus into patients with and without DKA and investigated the multiple proximal renal tubular functions and distal renal tubular functions simultaneously.

## Materials and methods

This retrospective study was conducted on Japanese patients newly diagnosed with type 1 diabetes mellitus who were admitted to the Department of Pediatrics at Fujita Health University Hospital or Fujita Health University Okazaki Medical Center between July 2018 and October 2023. Patients’ information and data were extracted from electronic medical records. The diagnosis criteria for DKA include hyperglycemia (blood glucose > 200 mg/dL), blood pH < 7.3 and/or HCO_3_^–^ < 15 mmol/L, and ketonuria and/or ketonemia [10]. Our participants were classified into those with and without DKA at the time of diagnosis. The first test results of blood and urine samples were recorded. Blood gas analyses were performed on venous blood. Urinary amino acid levels were measured on the first voided spot urine using liquid chromatography/mass spectrometry.

All statistical analyses were performed using EZR (Saitama Medical Center, Jichi Medical University, Saitama, Japan), which is a graphical user interface for R (The R Foundation for Statistical Computing, Vienna, Austria) [11].

The data were expressed as median values with interquartile ranges (IQRs) for skewed data. Mann–Whitney U test was used, with a p value of < 0.05 being considered statistically significant.

## Results

**Fig. 1:**
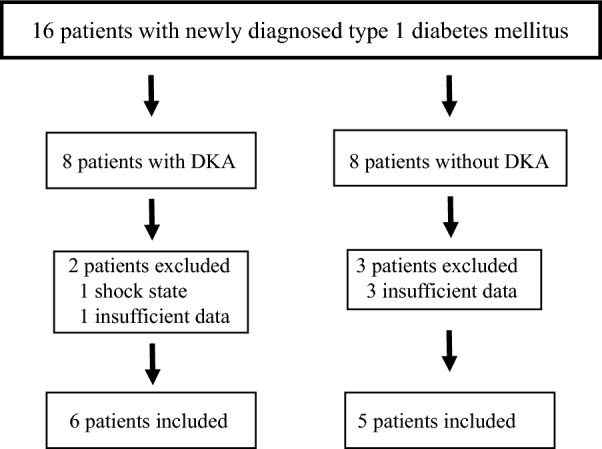
Flow diagram of the participant inclusion process. DKA, diabetic ketoacidosis

There were 16 Asian patients newly diagnosed with type 1 diabetes mellitus between July 2018 and October 2023 (Fig. 1). Eight of them had DKA and 8 did not have it at the time of diagnosis. Eleven patients were finally analyzed, as five were excluding from the study (one patient with shock and one patient with insufficient test results from those with DKA and three patients with insufficient test results from those without DKA).

**Fig. 2:**
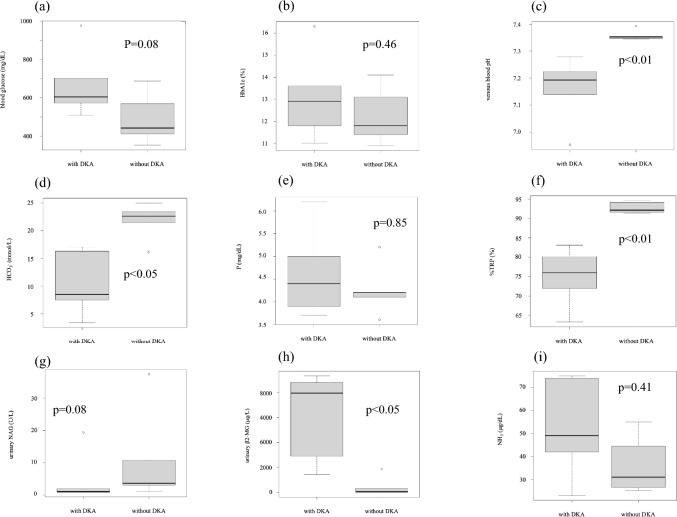
Comparison of laboratory findings presented in boxplots. The dots indicate the outliers in the box-and-whisker diagram. **a** Blood glucose (mg/dL), **b** HbA1c (%), **c** Venous blood pH, **d** HCO_3_^–^ (mmol/L), **e** P (mg/dL), **f** %TRP (%), **g** Urinary NAG (U/L), **h** Urinary β2-MG, and **i** NH_3_ (μg/dL). *DKA* diabetic ketoacidosis, *%TRP* tubular reabsorption of phosphorus, *NAG* N-acetyl-beta-glucosaminidase, *β2-MG* β2-microglobulin

The profiles of our study participants are presented in Table 1. The male-to-female ratio was 6:5 in the entire study population, with 4:2 among patients with DKA and 2:3 among patients without DKA. The median age was 9.0 (5.5–10.0) years for all cases, and there was no statistically significant difference in this parameter between patients with and without DKA (p = 0.355). The median blood glucose levels were 573 mg/dL (475.5–657.5) in all patients and were higher in patients with DKA but was not statistically significant (p = 0.0823; Table 2, Fig. 2a). The median HbA1c was 12.5% (11.6–13.45) in all patients, and no significant difference was observed between patients with and without DKA (p = 0.464; Table 2, Fig. 2b). Venous blood gas analyses demonstrated that venous blood pH was 7.279 (7.193–7.350) in all patients and was significantly lower in patients with DKA (p = 0.004; Table 2, Fig. 2c). HCO_3_^–^ was 16.30 mmol/L (8.50–22.05) in all patients and was significantly lower in patients with DKA (p = 0.017; Table 2, Fig. 2d).

**Table 1:** Clinical characteristics of patients upon admission

	With DKA						Without DKA				
Patient	1	2	3	4	5	6	7	8	9	10	11
Age (year)	5	10	9	12	12	6	1	2	10	10	9
Sex	m	m	f	m	f	m	m	f	f	m	f
Autoandibodies	anti-GAD	anti-GAD	anti-GAD	anti-GAD	anti-insulin	anti-GAD anti-IA-2	anti-GAD anti-insulin anti-IA-2	anti-GAD anti-insulin anti-IA-2	anti-GAD	anti-IA-2	anti-GAD
Blood glusoce (mg/dL)	628	509	702	580	976	573	687	569	411	353	442
HbA1c (%)	11.8	16.3	13.6	13.3	11	12.5	11.4	10.9	11.8	13.1	14.1
NH_3_(μg/dL)	not done	49	42	23	75	74	55	28	not done	34	25
P (mg/dL)	3.7	4.7	5	3.9	6.2	4.1	5.2	4.2	3.6	4.2	4.1
Venous blood pH	7.196	7.139	7.189	7.224	6.952	7.279	7.345	7.347	7.392	7.352	7.355
HCO_3_^–^ (mmol/L)	7.5	7.8	9.2	17	3.4	16.3	21.5	22.6	23.4	16.1	25
Corrected HCO_3_^–^ (mmol/L)	20.0	27	25	27	28	20.7				27	
Urinary pH	5.0	5	5.5	5	5.5	5.5	6	6.5	5.5	5.5	5.5
Urinary NAG (U/L)	2.0	0.9	1.1	0.9	19.4	0.9	37.4	3.1	10.8	3.6	1.2
Urinary β2-MG (μg/L)	9352.7	8487.0	8821.0	1451	7419.0	2906.0	1874.0	63.0	50 >	334.0	50 >
Urinary glucose	4 +	4 +	4 +	4 +	4 +	4 +	4 +	4 +	4 +	4 +	4 +
Urinary keton bodies	3 +	4 +	4 +	3 +	3 +	4 +	not done	1 +	3 +	4 +	2 +
%TRP (%)(90.5–92.5)	72.0	80.2	63.3	79.8	72.1	83.1	91.4	94.6	92.1	91.6	94.2
Urinary amino acid	done	not done	done	done	done	done	done	done	done	done	done
TSH (μIU/mL)(0.500–4.800)	0.971	1.461	0.924	0.839	0.564	0.983	1.686	1.353	1.045	1.705	2.47
fT3 (pg/mL)(2.51–4.16)	1.42	4.21	1.51	1.04	1.19	2.08	2.39	3.12	3.77	2.9	3.23
fT4 (ng/dL)(0.83–1.77)	0.817	1.17	1.02	0.87	0.8	1.15	0.94	1.38	1.76	1.58	1.31

*m* male, *f* female, *DKA* diabetic ketoacidosis, *GAD* glutamic acid decarboxylase, *IA-2* insulinoma-associated protein-2, *NAG* N-acetyl-beta-glucosaminidase, *β2-MG* β2-microglobulin, *%TRP* tubular reabsorption of phosphorus

**Table 2:** Comparisons of characteristics and laboratory findings

		All patients	With DKA	Without DKA	P value
Sex male (%)		6/11 (54.5)	4/6 (66.6)	2/5 (40.0)	
			Median (IQR)	Median (IQR)	
Age (year)		9.0(5.5–10.0)	9.5(6.75–11.5)	9.0(2.0–10.0)	0.355
Blood glucose (mg/dL)		573(475.5–657.5)	604 (574.75–683.5)	442.0 (411.0–569.0)	0.0823
HbA1c (%)		12.5(11.6–13.45)	12.9 (11.975–13.525)	11.8 (11.4–13.1)	0.464
NH_3_ (μg/dL)		42.0(28.0–55.0)	49.0 (42.0–74.0)	31.0(27.25–39.25)	0.413
P (mg/dL)		4.2(4.00–4.85)	4.4 (3.95–4.925)	4.2 0(4.10–4.20)	0.854
Venous blood pH		7.279(7.1925–7.3495)	7.1925 (7.1515–7.217)	7.352 (7.347–7.355)	0.00433
HCO_3_^–^ (mmol/L)		16.30(8.50–22.05)	8.5 (7.575–14.525)	22.6 (21.5–23.4)	0.0173
Urinary NAG (U/L)		2.0(1.0–7.2)	1 (0.9–1.775)	3.6(3.1–10.8)	0.08
Urinary β2-MG (μg/L)		1874(198.5–7953)	7953 (4034.25–8737.5)	63 (0–334)	0.0135
%TRP (%)		75.95(83.1–91.85)	75.95 (72.025–80.1)	92.1 (91.6–94.2)	0.00433
TSH (μIU/mL)		1.014 (0.9593–1.60275)	0.924 (0.839–0.971)	1.686(1.353–1.075)	0.00794
fT3 (pg/mL)		2.235(1.4425–3.065)	1.42 (1.19–1.51)	3.12 (2.90–3.23)	0.00794
fT4 (ng/dL)		1.085(0.8875–1.3625)	0.87 (0.817–1.02)	1.38 (1.31–1.58)	0.0317

The data are expressed as median values with interquartile ranges. *DKA* diabetic ketoacidosis, *IQR* interquartile range, *NAG* N-acetyl-beta-glucosaminidase, *β2-MG* β2-microglobulin, *%TRP* tubular reabsorption of phosphorus

### Investigation of proximal tubular function

Based on the corrected HCO_3_^–^ level, two of the patients with DKA were in both AG metabolic acidosis and normal AG metabolic acidosis (patients 1 and 6; Table 1). The median serum phosphorus level was 4.2 mg/dL (4.00–4.85) in all patients, and there was no significant difference in this parameter between patients with and without DKA (p = 0.854; Table 2, Fig. 2e). The tubular reabsorption of phosphorus rate was 75.95% (83.1–91.85) in all patients, and a significant decrease was observed in patients with DKA (p = 0.0043; Table 2, Fig. 2f). Urinary NAG level was 2.0 U/L (1.0–7.2) in all patients, and there was no statistically significant difference in this parameter between patients with and without DKA (p = 0.08; Table 2, Fig. 2g). The median urinary β2-MG level was 1874 μg/L (198.5–7953) in all patients and was significantly higher in patients with DKA (p = 0.0135; Table 2, Fig. 2h). Urinary amino acid level analyses demonstrated that the excretion of glutamic acid in patients with DKA was lower than that in patients without DKA (p < 0.05). Although the excretion of threonine, serine, asparagine, valine, leucine, isoleucine, and arginine in patients with DKA was higher than the reference value [12], there was no significant difference in this parameter between patients with and without DKA (Table 3).

**Table 3:** Comparisons of amino acid excretion

		All patients	With DKA	Without DKA	P value
amino acid	reference value	median (IQR)	median (IQR)	median (IQR)	
	μmol/mgCr				
Taurine		1257.95 (583.075–1758.1)	616.9 (571.8–1799.2)	1285.6 (1230.3–1593.7)	1
Aspartic acid	〜0.22	0 (0–0)	0 (0–0)	0 (0–0)	1
Hydroxyproline	〜0.27	0 (0–0)	0 (0–0)	0 (0–72.9)	0.18
Threonine	〜0.59	582 (227.95–1674.625)	2005.3 (501.5–2269.1)	285.1 (208.9–662.5)	0.31
Serine	0.23〜1.39	1438.2 (714.725–2568.925)	2618.2 (873.8–4449.1)	933.2 (661.7–1943.2)	0.421
Asparagine	〜1.04	615 (216.85–1422.925)	1631.8 (560–2035.8)	275.8 (197.2–670)	0.31
Glutamic acid	〜0.43	7.25 (0–30.575)	0 (0–0)	31.1 (29–74.7)	0.0449
Glutamine	〜1.57	528.35 (264.1–805.425)	340.9 (238.5–840.4)	625.6 (431.1–700.5)	0.69
Sarcosine		0 (0–0)	0 (0–0)	0 (0–0)	
α-Aminoadipic acid		87.62 (68.525–137.075)	97.5 (77.3 – 180.7)	77.8 (65.6–125)	0.69
ProIine	〜0.16	0 (0–0)	0 (0–0)	0 (0–16.05)	0.424
Glycine	0.91〜4.87	1375.7 (756.85–2643.525)	950.9 (302.3–2214)	1800.5 (790–2810)	0.421
Alanine	〜1.72	644.3 (317–1262.475)	765.5 (523.1–1414)	349.7 (306.1–807.9)	0.841
CitrulIine	〜0.15	66.45 (43–186.975)	90 (51.5–219.3)	51.1(40.3–81.4)	0.69
α-Aminobutyric acid		74.1 (32.875–116.275)	101.8 (87.7–140.8)	46.6 (28.3–60.5)	0.222
Valine	〜0.21	290.25 (194.725–465.85)	484.2 (410.8–490.9)	227.2 (183.9–276.8)	0.0952
Cystine	〜0.18	140.1 (97.8–173.75)	119.1 (88.9–164.9)	154.6 (125.6–186.3)	0.31
Cystathionine	〜0.06	0 (0–0)	0 (0–0)	0 (0–13.1)	0.18
Methionine	〜0.17	31.25 (19–36.6)	37.7 (18.6–67.3)	30.4 (20.2–32.1)	0.548
Isoleucine	〜0.09	79 (59.275–137.2)	137.3 (136.9–138.3)	66.7 (56.8–76.4)	0.0952
Leucine	〜0.20	238.4 (141.75–401.925)	413.6 (366.9–507.5)	161.7 (135.1–226.8)	0.0952
Tyrosine	〜0.73	232.9 (179.9–413.95)	307.3 (226.2–492.5)	181.4 (179.4–239.6)	0.421
Phenylalanine	〜0.29	139.35 (93.175–240.875)	200.9 (146.2–320)	100.6 (90.7–132.5)	0.31
r-Amino β-hydroxybutyric acid		0 (0–0)	0 (0–0)	0 (0–0)	
β-AIanie		21.4 (0–40.425)	39.3 (0–40.8)	20.0 (0–22.8)	0.666
β-Amino-iso-butyric acid		552.4 (169.65–1055.625)	152.3 (147.4–907.5)	592.1 (512.7–1689.4)	0.222
γ-Aminobutyric acid		0 (0–0)	0 (0–0)	0 (0–0)	
Homocystine		0 (0–0)	0 (0–0)	0 (0–0)	
Histidine	0.26〜4.10	2793.2 (1583.15–3703.9)	3176.4 (2193.8–6671.7)	1760 (1524.2–3392.6)	0.31
3-Methylhistidine		293.45 (179.3–343.35)	334.2 (316.2–346.4)	226.1 (163.7–270.7)	0.421
1-Methylhistidine		137.5 (102.375–405.525)	105 (101.5–491.2)	141.1 (133.9–148.5)	1
Tryptophan		241.35 (108.25–266.15)	3716.4 (2193.8–6671.7)	112.9 (106.7–257.9)	0.548
Hydroxylysine	〜0.09	18.7 (3.05–26.2)	0 (0–28.2)	18.9 (18.5–20.2)	0.526
Ornithine	〜0.12	50.6 (32.05–74.125)	42.3 (19.3–77.3)	58.9 (37.6–64.6)	0.841
Lysine	〜0.59	626 (401.275–859.025)	589.5 (203.8–893.6)	662.5 (456.1–755.3)	0.841
Arginine	〜0.11	85.3 (54.575–139.6)	140 (63.8–152.6)	83.9 (51.5–86.7)	0.421

The data are expressed as median values with interquartile ranges. *DKA* diabetic ketoacidosis, *IQR* interquartile range, *mgCr* mg creatinine

### Investigation of distal tubular function

In the presence of metabolic acidosis, no patients with or without DKA demonstrated a urine pH above 5.5, suggesting that there was no apparent impaired urinary acidification (Table 1). No patients demonstrated NH_3_ level elevation in either group. The median NH_3_ level was 42.0 (28.0–55.0) μg/dL in all patients, and this parameter did not differ significantly between patients with and without DKA (p = 0.413; Tables 1 and 2, Fig. 2i).

In patient number 2, 5, and 6 (Table 1), who experienced DKA as a complication, proximal renal tubular functions were re-evaluated after recovery from DKA. Re-evaluation was performed on days 9, 23, and 30 after admission and demonstrated that urinary β2-MG levels were 57.0, 107.0, and 130.0, and tubular reabsorption of phosphorus were 94.2%, 93.8%, and 93.4%, respectively, suggesting that proximal renal tubular dysfunction was transient.

### Investigation of thyroid function

**Fig. 3:**
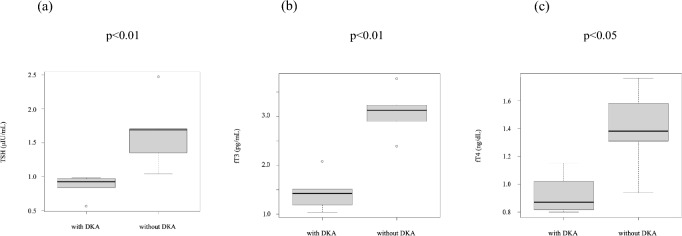
Comparison of thyroid function. **a** TSH (μIU/mL), **b** fT3 (pg/mL), **c** fT4 (ng/dL). *DKA* diabetic ketoacidosis

Thyroid function was compared. The median TSH, fT3, and fT4 levels were 1.014 (0.9593–1.60275) μIU/mL, 2.235 (1.4425–3.065) pg/ml, and 1.085 (0.8875–1.3625) ng/dL, respectively in all patients, and were significantly lower in patients with DKA (p < 0.001, p < 0.001, p = 0.0317, respectively; Table 2, Fig. 3). Thyroid autoantibodies were not measured.

## Discussion

In this study, high urinary β2-MG levels and low tubular reabsorption of phosphorus were observed in all patients with DKA at the time of diagnosis of type 1 diabetes mellitus, and increased urinary excretion of some amino acids was also observed. Since multiple proximal renal tubular dysfunctions were observed, patients with DKA were thought to present with Fanconi syndrome. In all three patients in whom proximal renal tubular functions were re-evaluated after the recovery from DKA, urinary β2-MG levels and tubular reabsorption of phosphorus improved within one month, suggesting that the impaired proximal renal tubular functions were transient, and this was due to DKA. Fanconi syndrome consists of multiple proximal renal tubular dysfunctions, resulting in proximal renal tubular acidosis, increased urinary amino acid excretion, impaired phosphorus reabsorption, increased urinary β2-MG, and electrolyte abnormalities [8]. To date, it is known that DKA is associated with normal AG metabolic acidosis [4], transient high urinary β2-MG [6], increased urinary amino acid excretion [7, 8], high urinary NAG, and decreased phosphorus reabsorption [6]. However, these phenomena were investigated separately. At the onset of childhood type 1 diabetes mellitus, some kind of proximal renal tubular damage was observed in all patients with DKA; moreover, even in the patients without DKA, some kind of proximal tubular damage was frequently observed [9]. These proximal renal tubular damages were short-lived. These findings strongly suggest that in all patients with DKA, multiple proximal renal tubular dysfunctions occur, resulting in Fanconi syndrome, which is a transient condition. We previously reported a case of newly diagnosed type 1 diabetes mellitus with DKA that presented with transient Fanconi syndrome [13]. Although this study involved a small number of patients, we investigated multiple proximal tubular functions simultaneously in the same patient. As a result, all patients with newly diagnosed type 1 diabetes mellitus with DKA had multiple proximal renal tubular dysfunctions, which were alleviated after recovery from DKA. These findings suggest that newly diagnosed type 1 diabetes mellitus with DKA is generally complicated with transient Fanconi syndrome due to DKA. Moreover, the fact that there is a difference in proximal renal tubular dysfunction between patients with and without DKA suggests that the diagnostic criteria for DKA imply the pathophysiological factors that may cause proximal renal tubular dysfunction. Classifying type 1 diabetes mellitus based on DKA criteria might be appropriate in the view of proximal renal tubular dysfunction.

In this study, tubular reabsorption of phosphorus was decreased in patients with DKA; however, no patients demonstrated hypophosphatemia. According to previous reports, hypophosphatemia occurs between the diagnosis and treatment of DKA, which results from an absolute lack of phosphorus in the whole body due to metabolic disturbance before the onset of DKA, an increase in phosphorus uptake into cells due to insulin treatment, and the loss of phosphorus to urine due to osmotic diuresis [14]. It has been reported that the tubular reabsorption of phosphorus decreases in patients with long-term poorly controlled type 1 diabetes mellitus [6]. However, the present study investigated patients newly diagnosed with type 1 diabetes mellitus in whom the decrease in tubular reabsorption of phosphorus was not due to long-term morbidity. According to the present study, tubular reabsorption of phosphorus is decreased in patients with DKA, suggesting that proximal renal tubular dysfunction is also involved in hypophosphatemia during DKA.

In the present study, two patients with DKA were suspected to have normal AG metabolic acidosis as complication. In general, normal AG metabolic acidosis during DKA is thought to be caused by the excretion of HCO_3_^–^ into urine and lungs. It is metabolized from the ketone bodies generated during DKA [5]. The present study demonstrated that there are multiple proximal tubular dysfunctions during DKA, which implies that impaired HCO_3_^–^ reabsorption in the proximal tubule could occur, resulting in proximal renal tubular acidosis and normal AG metabolic acidosis. The bicarbonate loading test is necessary to diagnose impaired HCO_3_^–^ reabsorption. Since proximal renal tubular dysfunction during DKA is transient and in short period, it is difficult to perform bicarbonate loading test during DKA. Therefore, it is difficult to prove directly whether proximal renal tubular acidosis occurs during DKA. There is a case report of transient proximal renal tubular acidosis for seven weeks after DKA [15], suggesting that proximal renal tubular dysfunction may be involved in normal AG metabolic acidosis during DKA.

In this study, although including a small number of patients, there was no statistically significant difference between patients with and without DKA in urinary amino acid excretion; however, the excretion of some amino acids exceeded the reference value in patients with DKA. To our knowledge, there were two reports that investigated amino acid excretion in urine during DKA hitherto [7, 8]. The excretion of branched-chain amino acids (valine, leucine, and isoleucine), histidine, serine, and threonine into the urine during DKA was increased. On the contrary, the excretion of glutamic acid, glutamine, glycine, and taurine was decreased [7]. The influence of amino acid metabolism associated with DKA was suggested in this difference. Moreover, the excretion of some amino acids such as asparagine and histidine was strongly correlated with urinary β2-MG excretion, suggesting the influence of proximal renal tubular dysfunction [7]. It has been also reported that the excretion of histidine, threonine, tryptophan, and leucine was increased during DKA and that they decreased over time and reached their lowest levels at three months, indicating a relationship with proximal renal tubular dysfunction [8]. In the present study, the asparagine level, which is suggested to be associated with proximal renal tubular dysfunction during DKA, exceeded the reference value, which implied proximal renal tubular dysfunction. It is necessary to elucidate the relationship between proximal renal tubular dysfunction and the amino acid metabolism of DKA in urinary amino acid excretion.

The mechanism underlying transient Fanconi syndrome during DKA is thought to be reduced glucose uptake into renal tubular cells due to insulin deficiency, leading to decreased ATP production, which results in energy deficiency and renal tubular dysfunction [16, 17]. Moreover, during DKA, lipolysis is accelerated and the blood free fatty acids (FFA) level is increased. Glucagon excess accelerates the conversion of FFAs to ketone bodies, resulting in an increase in blood ketone body levels and also an increase in urinary ketone excretion. Ketone bodies may directly damage renal tubular cells [18], contributing to renal tubular dysfunction. It has been reported that hyperglycemic states such as DKA induce proximal tubular degeneration [19]. Notably, herein, blood glucose levels were higher in patients with DKA who had proximal renal tubular dysfunction than in those without DKA, although it was not statistically significant, implying the existence of an association between high glucose levels and proximal renal tubular dysfunction. Treatment with insulin improves glucose uptake into renal tubular cells, restores ATP production, and decreases ketone body production, resulting in the relief of renal tubular dysfunction. Therefore, Fanconi syndrome is thought to be transient. In fact, in the present study, in all patients with DKA, renal tubular dysfunction was alleviated shortly after insulin treatment, which supports this mechanism.

In the present study, no patients with DKA demonstrated urinary pH values in excess of 5.5 even in the presence of metabolic acidosis, which implied no urinary acidification impairment. Moreover, no increase in NH_3_ was observed. These findings suggest that no patients presented with distal renal tubular acidosis. A few cases were reported to present with distal renal tubular dysfunction associated with type 1 diabetes mellitus, including distal renal tubular acidosis after long-term morbidity of diabetes mellitus [20, 21]. However, in all of the cases, distal renal tubular dysfunction was diagnosed without DKA. Moreover, when renal function declines due to diabetic nephropathy, distal renal tubular dysfunction as type 4 renal tubular acidosis may occur [22]. To the best of our knowledge, distal renal tubular function has never been investigated in newly diagnosed type 1 diabetes mellitus with DKA before, and the present study is the first to investigate distal renal tubular function. Although the present study was conducted in a small number of patients, distal renal tubular acidosis was less likely to occur as a complication of newly diagnosed type 1 diabetes mellitus with DKA.

At the onset of type 1 diabetes mellitus, autoimmune thyroid diseases may be present [23] and patients with DKA often present with low T3 syndrome [24, 25]. In the patients analyzed in this study, hypothyroidism, the so-called low T3 syndrome [25], was observed in patients with DKA compared with patients without DKA. Hypothyroidism can lead to distal renal tubular acidosis [26], which can result in secondary Fanconi syndrome if left untreated for a long period [27–29]. In patients with impaired reabsorption of multiple substances in the proximal tubules, it may take several months to several years to improve Fanconi syndrome secondary to distal renal tubular acidosis after treatment of the distal renal tubular acidosis [27, 29]. Herein, hypothyroidism was observed in the patient with DKA, however, distal renal tubular acidosis was not observed and Fanconi syndrome improved in a short period; therefore, hypothyroidism is not believed to be the cause of Fanconi syndrome.

The strong points of the present study are that multiple renal tubular functions were investigated simultaneously in the same patients with newly diagnosed type 1 diabetes mellitus with and without DKA. Therefore, the influence of long-term morbidity and other factors was minimal. The main limitation of the present study is that it was a single-center study involving only a small number of patients. Moreover, the influence of acute kidney injury was not considered.

## Conclusion

In patients with newly diagnosed type 1 diabetes mellitus with DKA, multiple proximal renal tubular dysfunctions are observed, suggesting transient Fanconi syndrome. Proximal renal tubular dysfunction is suggested to be involved in the pathogenesis of hypophosphatemia and metabolic acidosis during DKA. Distal renal tubular dysfunction is unlikely. The diagnostic criteria for DKA are appropriate also in the view of proximal renal tubular dysfunction and are considered to suggest pathophysiological factors that may cause proximal renal tubular dysfunction.

## Data Availability

No datasets were generated or analysed during the current study.
